# High abundance and expression of transposases in bacteria from the Baltic Sea

**DOI:** 10.1038/ismej.2017.114

**Published:** 2017-07-21

**Authors:** Theoden Vigil-Stenman, Karolina Ininbergs, Birgitta Bergman, Martin Ekman

**Affiliations:** 1 Department of Ecology, Environment and Plant Sciences, Stockholm University, Stockholm, Sweden

## Abstract

Transposases are mobile genetic elements suggested to have an important role in bacterial genome plasticity and host adaptation but their transcriptional activity in natural bacterial communities is largely unexplored. Here we analyzed metagenomes and -transcriptomes of size fractionated (0.1–0.8, 0.8–3.0 and 3.0–200 μm) bacterial communities from the brackish Baltic Sea, and adjacent marine waters. The Baltic Sea transposase levels, up to 1.7% of bacterial genes and 2% of bacterial transcripts, were considerably higher than in marine waters and similar to levels reported for extreme environments. Large variations in expression were found between transposase families and groups of bacteria, with a two-fold higher transcription in Cyanobacteria than in any other phylum. The community-level results were corroborated at the genus level by *Synechococcus* transposases reaching up to 5.2% of genes and 6.9% of transcripts, which is in contrast to marine *Synechococcus* that largely lack these genes. Levels peaked in *Synechococcus* from the largest size fraction, suggesting high frequencies of lateral gene transfer and high genome plasticity in colony-forming picocyanobacteria. Together, the results support an elevated rate of transposition-based genome change and adaptation in bacterial populations of the Baltic Sea, and possibly also of other highly dynamic estuarine waters.

## Introduction

Mobile genetic elements are represented in their simplest form by insertion sequences (ISs), ~1000 base pair (bp) DNA sequences consisting of an open reading frame (ORF) encoding a transposase, flanked by inverted repeats. The transposase protein mediates a process known as transposition, where an IS is excised or copied from one location in the genome and inserted at another.

The abundance of transposase genes in prokaryotes is highly variable, ranging from zero to over a thousand copies per genome (Vigil Stenman, 2015). Transposases facilitate genomic rearrangements and gene duplications, either by incorporating adjacent genes during transpositions, or by enabling genetic recombination by being present in multiple copies. They may also influence expression of host genes (Casacuberta and González, 2013), including via regulatory RNAs (Ellis and Haniford, 2016). Transposases furthermore move between genomes via mechanisms of horizontal gene transfer (HGT), and may promote transfer of functional genes (that is, as transposons) (Frost *et al.*, 2005). They thus represent potent means of adaptation, explaining their high abundance in organisms inhabiting stressful or extreme environments (Li *et al.*, 2014; Nelson *et al.*, 2011; Leduc and Ferroni, 1994), and why transposition events are induced by cell stress (Casacuberta and González, 2013).

Transposases may also pose a threat to an organism as the sequence specificity of transposase insertions is usually low, and transpositions may disrupt vital genes. This may explain why larger genomes on average have a higher frequency of transposases (Touchon and Rocha, 2007), since they contain more neutral insertions sites, such as, redundant or noncoding regions, lacking critical cellular functions. For example, transposases often constitute over 2% of the large genomes of filamentous Cyanobacteria but are mostly absent from small-genomed marine unicellular picocyanobacteria (*Synechococcus* and *Prochlorococcus*) (Vigil-Stenman *et al.*, 2015). Likewise, the relaxed selection pressure offered by a symbiotic host may allow the higher transposase content often seen in bacteria that recently adopted a symbiotic or pathogenic lifestyle (Walker and Langridge, 2008; Ran *et al.*, 2010; Vigil-Stenman *et al.*, 2015). However, the highest transposase frequencies recorded are from the free-living Cyanobacteria *Microcystis aeruginosa* (Kaneko *et al.*, 2007), and *Crocosphaera watsonii* (Bench *et al.*, 2011), 14 and 23% of their genomes, respectively. As these organisms do not display features typical of known transposase-rich organisms, additional factors may influence transposase abundances in bacterial genomes.

Recent metagenomic surveys have analyzed transposase distribution in natural marine microbial populations. In surface waters of the open ocean most bacteria have few transposases (Li *et al.*, 2014), probably due to genomic streamlining. The transposase abundance increases in deep and low oxygen waters (DeLong *et al.*, 2006; Konstantinidis *et al.*, 2009), and also among ‘attached’ bacteria, either in biofilms (up to 8% of protein coding genes) (Brazelton and Baross, 2009), or associated with particles (up to 2%, Ganesh *et al.*, 2014). The depth dependency may be due to low growth rate and small effective population sizes (DeLong *et al.*, 2006), or to increasing particle abundance (Ganesh *et al.*, 2014). Higher abundance of transposases in attached bacteria may relate to higher rates of DNA exchange in dense populations and to the variable environment of particles selecting for transposase-mediated adaptability of the bacterial host (Ganesh *et al.*, 2014). Particles may thus function as hotspots for gene exchange and microbial evolution (Stewart, 2013). However, most studies of transposases in natural bacterial population are limited to gene abundance and it is mostly unclear how these abundances translate into actively transcribed transposase genes. Transposition is known to be tightly regulated including at the transcriptional level (Ellis and Haniford, 2016), but transposase gene expression may also be induced by environmental factors (Jäger *et al.*, 2009; Steffen *et al.*, 2014; Wemheuer *et al.*, 2015). Furthermore, data on transposase abundance in aquatic environments are mostly from open oceans while our knowledge on their role in coastal or estuarine waters, where 50% of the bacterial population may be associated with particles (Crump *et al.*, 1998), is rudimentary. Estuaries are also more variable ecosystems (for example, bioavailability of nutrients, light intensity, trace metals and salinity) and therefore more demanding in terms of adaptability of the bacterial community.

**Figure 1:**
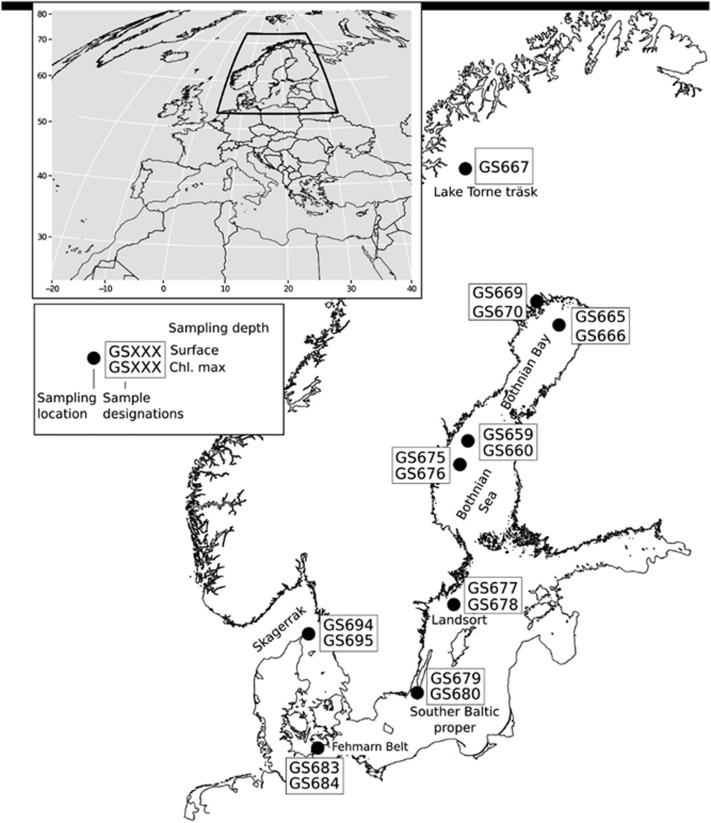
Geographic locations of the sites sampled in the Baltic Sea and adjacent limnic and marine waters. The sites are denoted GS followed by a number, from GS667 in Lake Torne Träsk in the north, through the brackish water Baltic Sea basins to the marine west coast of Sweden at GS694/695. The sampling embraced microbes in surface waters and at the chlorophyll maximum depth (4–19 m depth), as well as a sample from 74 m depth at GS678 at the Landsort Deep site.

To expand our understanding of the role of transposases in marine bacterial populations we analyzed metagenomes and metatranscriptomes from the Baltic Sea. This semi-enclosed water body can be considered the common estuary of several major rivers that flow into it, creating a salinity gradient ranging from near 0 in the north to around 15 PSU (practical salinity units) in the south. Via the narrow Danish straits, the Baltic Sea connects to Kattegat and the fully marine Skagerrak (30 PSU) (Figure 1). Limited water exchange results in long water retention times (>5 years; Wulff and Stigebrandt, 1989), allowing bacterial communities to adapt. For example, picocyanobacteria with unusual light adaptation capacities were recently identified in these waters (Larsson *et al.*, 2014). The Baltic Sea is also influenced by anthropogenically inflicted eutrophication (Cederwall and Elmgren, 1990) leading to the spreading of anoxic bottom waters and is rich in particulate matter originating from river drainage and phytoplankton, including filamentous cyanobacteria forming massive blooms. Influenced by such processes a range of gradients in salinity, nutrients and oxygen are acting on the microbial life, as are large seasonal fluctuations in light (0–24 h of daylight) and temperature (−0.3 to 20 °C); making the Baltic Sea an exceptionally dynamic environment and a model habitat to study the role of transposases in bacteria.

## Materials and methods

### Sample collection and sequencing

The Baltic Sea samples were collected in July 2009 from seven geographical locations along a 1800 km long transect encompassing a north–south salinity gradient within the Baltic Sea and neighboring water bodies (Figure 1) (Dupont *et al.*, 2014). At each station, samples were collected from surface waters (0.3 m depth), and from the subsurface chlorophyll maximum depth (5–19 m), except for the freshwater lake Torne Träsk (station GS667), where only a surface water sample was collected, and Landsort Deep, where one sample was collected from the chlorophyll maximum level and one from the suboxic zone (70 m depth). The water samples were serially filtered into three cellular size fractions (0.1–0.8, 0.8–3.0 and 3.0–200 μm) as described in Allen *et al.* (2012).

Sample preservation, extraction and sequencing of the metagenome were conducted as described previously (Allen *et al.*, 2012; Dupont *et al.*, 2014; Asplund-Samuelsson *et al.*, 2016). Sequencing was performed at J Craig Venter Institute, La Jolla, California, USA. Reads were annotated using the JCVI metagenomic annotation pipeline (Tanenbaum *et al.*, 2010), along with APIS (Allen *et al.*, 2012), and fragment recruitment (Rusch *et al.*, 2007). A global assembly of all sequence data was performed using the Newbler Assembler (Margulies *et al.*, 2005), with a final assembly of 490 million bp, a N50 of 1.5 Kbp and a largest contig of 106 Kbp (Dupont *et al.*, 2014). Sample preservation and extraction of the metatranscriptomes was previously described in Asplund-Samuelsson *et al.* (2016). RNA quality was analyzed on a 2100 Bioanalyzer (Asplund-Samuelsson *et al.*, 2016). The transcriptome was sequenced from cDNA using Illumina sequencing, and quality trimmed and searched against 16 S ribosomal RNA and vectorDB to remove artifacts and ribosomal RNA sequences. Reads were assembled into transcript contigs using CLC Genomics Workbench (v. 6) (Qiagen, Germantown, MD, USA), ORFs were called with fraggenescan, and the reads were then mapped back to the assembled transcript ORFs using CLC. ORFs were annotated by blastp vs PhyloDB and hmmscan with Pfam/TIGRFAM (Asplund-Samuelsson *et al.*, 2016). At some locations, metagenome and transcriptome samples were not collected at the same sites (Bothnian Bay and Bothnian Sea), but at neighboring sites with comparable salinities. Additional details on the Baltic Sea DNA and RNA samples and processing, and resulting data sets are described in Dupont *et al.* (2014) (for metagenomes) and Asplund-Samuelsson *et al.* (2016) (for metatranscriptomes). The CalCOFI samples were collected and sequenced as described in Dupont *et al.* (2015).

### Identification and normalization of transposases

Identification of transposases was performed by using BlastX to compare the nucleotide sequences of metagenomic and transcriptomic contigs to transposase amino-acid sequences from the ISfinder (Siguier *et al.*, 2006) repository of annotated ISs. About 5173 amino-acid sequences from transposase ORFs were downloaded (1 May 2015) and manually curated to remove passenger genes and other amino-acid sequences not representing transposase proteins. Contigs from metagenomes or -transcriptomes were initially blastX:ed against the transposase amino-acid sequences, and all sequences matching transposase ORFs with an expect value of <10^−6^ were collected and their location marked. The search frequently produced several hits over the same nucleotide stretch, and these hits were used to designate a ‘footprint’, which was then searched again to find the most likely transposase(s) occupying the stretch. In this second blastX search, the transposase with the highest score was considered most similar to the occupying transposase, still with a requirement of expect value <10^−6^. In cases where the highest score transposase didn't encompass the entire footprint, the process was repeated until the entire footprint had been identified. After collection of all identified transposase hits, only those making up at least 30% of a transposase ORF amino-acid sequence were considered to be transposases. The amount of transposases found for different stations and filter sizes are expressed as a quotient of all transposase reads divided by the total number of bacterial reads where no other normalization is specified. To assess the number of transposases per genome, transposase read counts were normalized by dividing the number of transposase reads by average number of hits to 35 common (Liu *et al.*, 2012) single-copy Cluster of Orthologous Groups of proteins (Supplementary Information Table 1).

### Statistics and data processing

To process the large amount of data generated by the meta-omic sequencing and subsequent transposase identification, genomic and transcriptomic contigs were stored in an SQLite3 (Hipp *et al.*, 2015) database along with transposase search data. Relevant data were extracted and analyzed using R 3.2.1 (R Core Team, 2015) together with the R libraries DBI and RSQLite. Figures were generated with the R libraries ggplot2 and rworldmap. Statstical tests for geographic and size fraction-based transpoase differences were carried out with the R function wilcox.test, and the linear models in Figure 7 were generated with the R function lm.

## Results

The metagenomic and -transcriptomic data sets analyzed were obtained from samples collected along a north–south transect of the brackish Baltic Sea (Figure 1; sampling stations GOS669–GOS684), with the freshwater Lake Torne Träsk (GOS667) constituting the northernmost sampling point, and from the marine waters of Skagerrak (GOS694 and GOS695). At most locations, samples were collected from two depths; surface and chlorophyll maximum (4–19 m depth) and serially filtered into three size fractions, denoted ‘small’ (0.1–0.8 μm), ‘medium’ (0.8–3.0 μm) and ‘large’ (3.0–200 μm) (Dupont *et al.*, 2014; Asplund-Samuelsson *et al.*, 2016).

### Abundance of transposase genes and transcripts

**Figure 2:**
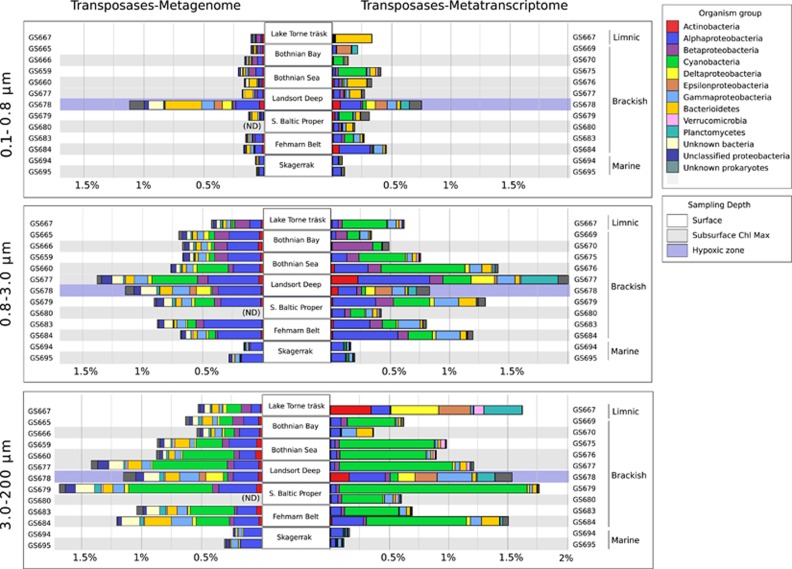
Transposase gene abundances and transcript levels in the Baltic Sea, Lake Torne Träsk and the marine Skagerrak. The graphs show transposase genes as a fraction of the total number of bacterial genes (left bars; metagenome) and transposase transcripts as a fraction of the total number of bacterial transcripts (right bars; metatranscriptome) at each station and for each of the three size fractions. Colors of bars denote organism groups, while gray bars represent sampling depth; surface (0.3 m), chlorophyll maximum (5–19 m) and the suboxic sample at Landsort Deep (74 m). Metagenomic samples from GS680 were not sequenced. Some metagenomes and metatranscriptomes were not generated from the same samples but from samples at adjacent locations, as seen in sample codes.

In Baltic Sea samples, transposases made up 0.11–1.7% of all bacterial metagenomic reads, and transposase transcripts 0.13–2.0% of all bacterial metatranscriptomic reads (Figure 2). The relative numbers of transposases varied depending on size fraction, sampling station and depth. In both the metagenome and -transcriptome, transposases were significantly (Wilcoxon *P*<0.001) less abundant in the small size fraction compared to the medium and large fractions. This difference was seen throughout the transect, except for the samples collected from deep hypoxic waters (74 m depth) at Landsort Deep (GS678), where the small size fraction contained high transposase levels, similar to those found in the two larger fractions.

Along the Baltic Sea sampling transect, the highest transposase gene and transcript levels were found in the central and southern Baltic Sea (Figure 2). The transposase level in the Baltic Sea (GS665–684) was furthermore significantly higher than in the marine Skagerrak (GS694–95), both in terms of gene abundance (median 0.7 vs 0.2% Wilcoxon’s *P*=0.008) and transcription (median 0.8 vs 0.1% Wilcoxon’s *P*<0.001). For an additional comparison of transposase abundances in the brackish Baltic Sea with a marine habitat, transposase frequencies in metagenomes and -transcriptomes from the fully marine, Pacific ocean (Dupont *et al.*, 2015) were determined. Here transposase abundances averaged only about 0.02% in the metagenomes and 0.002% in the metatranscriptomes (Supplementary Information Figure 1).

### Taxonomic affiliations

**Figure 3:**
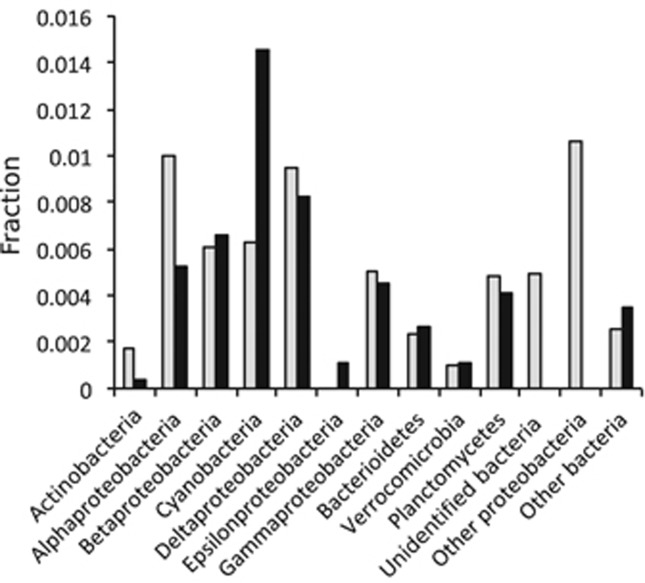
Transposase abundance/transcription in Baltic Sea bacterial phyla/classes. The graph shows median transposase gene (gray bars) and transposase transcript (black bars) abundance in phyla or classes. Bar heights represent numbers of transposase reads/transcripts annotated to an individual phylum, normalized to all reads/transcripts of the corresponding phylum. For example, the value corresponding to the cyanobacterial transcript abundance was calculated by dividing the number of cyanobacterial transposase transcripts with the total number of cyanobacterial transcripts.

On the basis of the taxonomic annotation of the metagenomic transposase reads, Alphaproteobacteria contributed most to the Baltic Sea transposase population (31%), followed by Cyanobacteria (11%) (Figure 2). This distribution partly reflects the Baltic Sea bacterial community composition, in which Alphaproteobacteria constitute the largest group (Supplementary Information Figure 2; Dupont *et al.*, 2014). However, normalizing at the phylum level (Figure 3) showed large differences in genomic transposase abundance among phyla, with high levels in Alpha- and Deltaproteobacteria, and low in Actinobacteria, Verrucomicrobia and Epsilonproteobacteria. Alphaproteobacterial transposases were mainly annotated to Rhodobacteriales and Rhizobiales, and few to the most abundant Alphaproteobacterial group, SAR11 (Supplementary Information Figure 3).

The taxonomic annotation of transposase transcripts contrasted the metagenome annotation. Here cyanobacterial transcripts were most abundant and constituted 29% of all transposase transcripts in the data set (Figure 2). Phylum level normalization showed that in Cyanobacteria the transposase transcripts constituted a considerably higher proportion of total transcripts than in any other major Baltic Sea bacterial phylum (Figure 3). The ratio between transposase transcripts and metagenomic transposase reads was 2.3 in Cyanobacteria, while in other bacterial phyla/classes it ranged from 1.2 (Verrucomicrobia) to 0.24 (Actinobacteria).

### Cyanobacterial transposases

**Figure 4:**
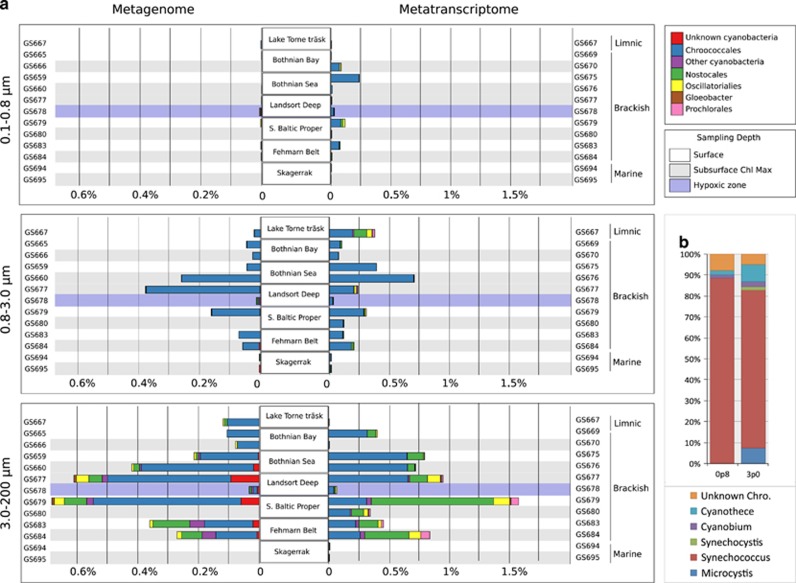
Cyanobacterial transposase genes and transcripts in the Baltic Sea and adjacent waters. (**a**) The horizontal bars represent cyanobacterial transposase sequences in metagenomes normalized to all bacterial sequences (left) and cyanobacterial transposase transcripts in metatranscriptomes normalized to all bacterial transcripts (right), in the three size fractions examined. Colors denote cyanobacterial orders (boxes, upper right). (**b**) Relative contribution by different genera to transposases annotated to the unicellular order Chroococcales.

**Figure 5:**
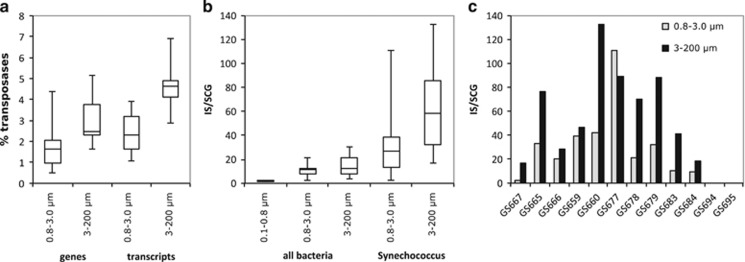
Transposases in the unicellular cyanobacterial genus *Synechococcus*. (**a**) Percentage of *Synechococcus* transposase genes (metagenomic reads) and transcripts (metatranscriptomic reads) of all *Synechococcus* genes and transcripts, respectively, in the two larger size fractions. (**b**) Number of transposase genes relative to average single-copy gene numbers in *Synechococcus* (right) and as reference also for all bacteria (left). (**c**) *Synechococcus* transposase genes relative to average single-copy gene numbers for each individual sample in the two larger size fractions along the Baltic Sea transect (Figure 1). Data from the small size fraction are not included due to the low number (2) of *Synechococcus* transposase reads detected.

The highest cyanobacterial transposase gene and transcript levels were found in central and southern Baltic Sea (Figure 4a), consistent with the distribution of cyanobacteria along the transect (Supplementary Information Figure 4). In contrast, cyanobacterial transposases were largely absent from the marine Skagerrak samples (GS694–95), despite a high abundance of cyanobacteria in these waters (Supplementary Information Figure 4). The cyanobacterial transposases were primarily annotated to the unicellular order Chroococcales (Figure 4a), which constituted 79% of the total Baltic Sea cyanobacterial population at the time of sampling (Supplementary Information Figure 4). The Chroococcales transposases in turn mostly belonged to the genus *Synechococcus* (83% Figure 4b), while few to *Cyanobium*, the second most abundant picocyanobacterial genus in the Baltic Sea (Supplementary Information Figure 5). Also transposases belonging to *Microcystis* were few (2% of Chroococcales transposases) while *Crocosphaera* transposases were not detected. Cyanobacterial transposase abundances (relative to all bacterial reads) increased with fraction size (Figure 4a) consistent with these bacteria having cell diameters >0.8 μm, and therefore primarily being captured on the two larger size filters (Supplementary Information Figure 4). For *Synechococcus* also the genomic abundance of transposases (that is, relative to all *Synechococcus* reads) increased with size fraction; from 1.6% in the medium to 2.4% in the large fraction (Wilcoxon’s *P*<0.01) (Figure 5a). In the metatranscriptomes, the corresponding numbers were 2.3% in the medium and 4.6% in the large fraction (Figure 5a). Transposases peaked in the central Baltic Sea and made up over 5.2% of all *Synechococcus* metagenomic reads at station GS679 and close to 7% of all gene transcripts at station GS677 (Supplementary Information Figures 6A and 6B). In the marine Skagerrak, no *Synechococcus* transposases were detected despite a *Synechococcus* population size similar to the Baltic Sea samples (Supplementary Information Figure 5).

Normalization to single-copy genes showed that *Synechococcus* from the medium size fraction contained on average 27 transposases per genome, while those from the large fraction had on average 58 (Wilcoxon’s *P*<0.01) (Figure 5b), reaching a maximum of 133 transposases per genome at station GS660 (Figure 5c). Per genome transposase numbers varied along the transect, and peaked in the central and southern Baltic Sea. The size fraction difference was however consistent at all sampling stations, except at GS677 (Figure 5c). Total reads relative to single-copy genes counts furthermore suggested no significant difference in genome size of *Synechococcus* from different size fractions and showed no clear geographic trend (Supplementary Information Figure 7).

To give perspective on our data, transposase numbers in genomes of *Synechococcus* reference strain were examined. While strains most closely related to the Baltic Sea population (Supplementary Information Figure 8), which mostly include strains isolated from coastal waters, contain between 9 and 50 transposase genes per genome (Table 1), reference strains related to the Skagerrak population, which all originate from open ocean waters, contain none.

### Transposase gene families

Screening for the presence of transposase families in the Baltic Sea (BS) metagenomes and -transcriptomes identified over 30 transposase families (Supplementary Information Figure 9). IS3 and IS5 were the over-all most abundant families in both the metagenomes and -transcriptomes, except for the large size fraction of the metatranscriptome, which was dominated by IS200/IS605 and IS256. IS200/IS605 was largely annotated to Nostocales with highest transcript levels detected in the southern Baltic Sea (GS679). IS256 transposases annotated to *Synechococcus* dominated transcription at sampling stations further north (GS675–77), and included the most highly transcribed transposase (0.04% of all transcriptome reads) in the data set. Most IS5 transcripts were also assigned to *Synechococcus*, while the IS3 family transcripts were largely proteobacterial.

**Figure 6:**
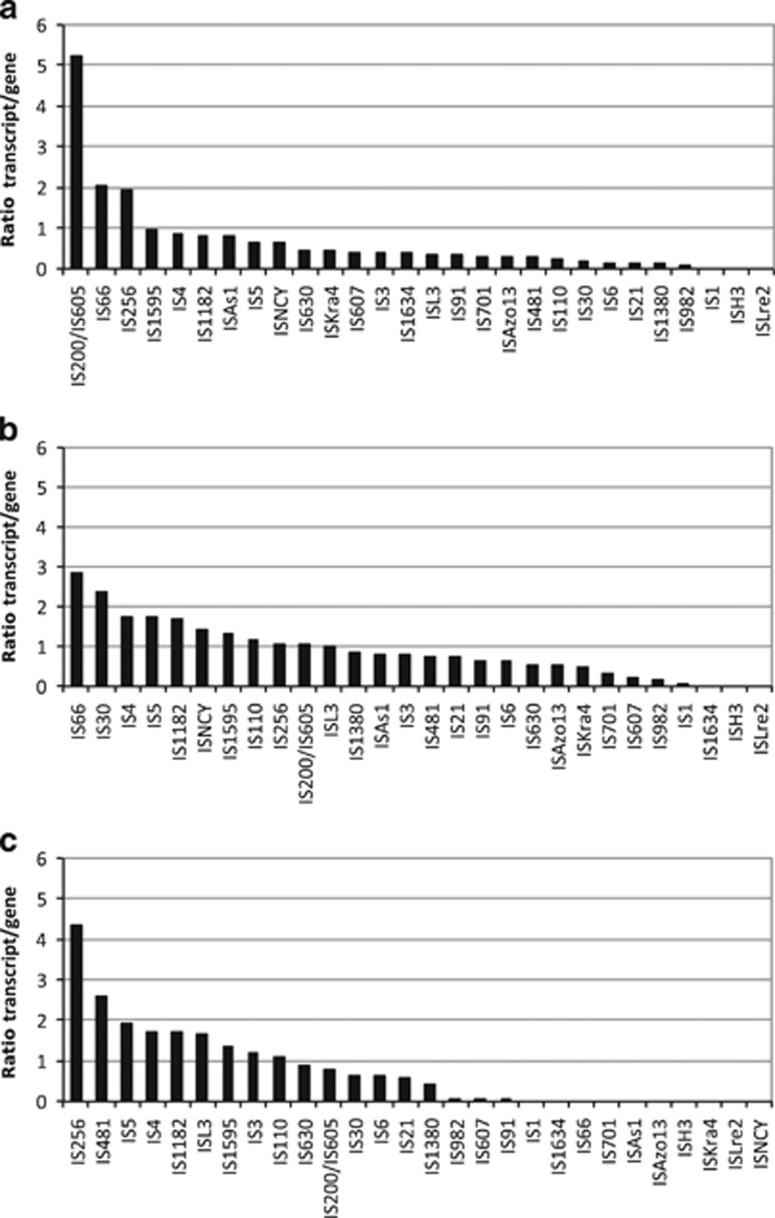
Transcriptional activity of transposase gene families in Baltic Sea bacteria. The transposase activity in (**a**) the large, (**b**) the medium and (**c**) the small size fraction is given as the medians of the ratios between transposase transcripts abundance (metatranscriptome) and transposase gene abundance (metagenome) in each sample.

Comparing the ratio between transcripts and metagenomic reads revealed large variations in transcriptional activity among the transposase families (Figure 6). The highest average ratio was found for IS200/IS605 in the largest size fraction, followed by IS66 and IS256 (Figure 6a). In the medium size fraction, the IS66 family showed the highest transcriptional activity, while IS200/605 and IS256 were considerably less active (Figure 6b). IS256 was again highly active in the small size fraction (Figure 6c). The relative contribution of bacterial phyla to the transposase families is shown in Supplementary Information Figure 10.

### Genome size, transposase abundance and transcription

**Figure 7:**
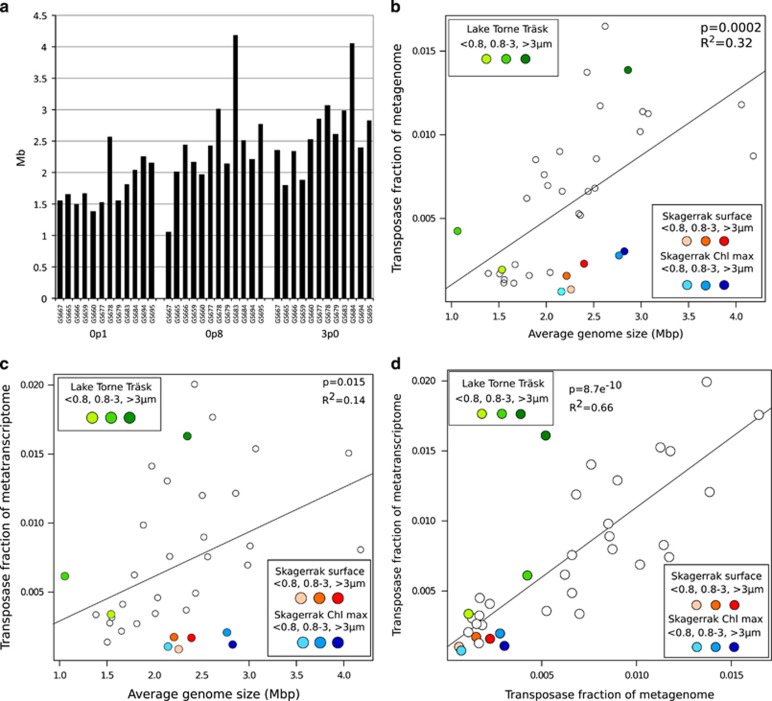
Genome size and transposase gene and transcript correlations. (**a**) Average genome size for each individual sample in the data set in the three size fractions. Data plotted from Dupont *et al.* (2014). (**b**) Correlation between average genome size and transposase gene fraction of metagenomes (Pearson’s *P*=8.7e−10, adjusted *R*^2^=0.66), and (**c**) transposase fractions of metatranscriptomes (Pearson’s *P*=0.015, adjusted *R*^2^= 0.14). Color codes given in boxes. (**d**) Correlation between transposase fraction of each metagenomic sample (*y* axis) and transposase fraction of each metatranscriptomic sample (*x* axis) (Pearson's *P*=8.7e−10, adjusted *R*^2^=0.66, *k*=1).

The average genome size in the Baltic Sea metagenomic samples was determined in a previous study (Dupont *et al.*, 2014). Further analysis of these data showed a weak trend of increasing genome sizes toward higher salinities, especially in the small size fraction (Figure 7a). The highest average genome size was found in bacteria from suboxic waters at Landsort Deep (74 m depth at GS678; Figure 7a). The genome size also correlated positively with transposase numbers, both in the metagenomes (Figure 7b) and the metatranscriptomes (Figure 7c). However, microbial samples from the fully marine Skagerrak deviated from this trend. With their comparatively large average genome sizes (2.2–2.8 Mb), their transposase content was half of that predicted using a linear genome size/transposase abundance model. Furthermore, transposase transcript abundance in the metatranscriptomic samples correlated positively with the transposase gene abundance in the metagenomic samples (Figure 7d).

## Discussion

Our data show that the transposase gene levels in Baltic Sea bacteria (0.11–1.7%, median 0.7%) are not only significantly higher than in bacteria from the marine waters examined here, but also in comparison to levels previously reported for both limnic (0.02–0.06%) and marine (0.03–0.2%) ecosystems (Li *et al.*, 2014). In fact, the transposase gene abundance in these brackish waters is similar to that of bacteria from ‘extreme’ environments, such as hot springs and saline lakes (0.4–2.5%, Li *et al.*, 2014). While small bacterial genome sizes may partly explain low transposase abundance in oligotrophic ocean habitats, it is noteworthy that genome size actually increased from the brackish Baltic Sea to the marine Skagerrak (Figure 7a). The Baltic Sea is a highly dynamic estuarine environment characterized by gradients in, for example, salinity, nutrients and oxygen, and is toward the south, where transposase levels peaked, also impacted by anthropogenic activities. The observed elevated transposase abundance/activity in the Baltic Sea may therefore rather be the result of positive selection for a higher adaptive capacity in a variable and/or stressful environment. Several studies have indeed found that transposition is induced by stress and environmental alterations including nutrient availability (Ilves *et al.*, 2001), temperature (Ohtsubo *et al.*, 2005) and UV light exposure (Eichenbaum and Livneh, 1998) (see also Wessler, 1996 and Capy *et al.*, 2000).

Transposition is however also known to be tightly regulated, including at the level of transcription, and is assumed to be kept at a minimum to reduce genome damage (Nagy and Chandler, 2004). In this context, our finding that up to 2% of all transcripts in Baltic Sea bacteria were transposases is unexpected. High transposase gene expressions have been recorded previously, reaching up to 15.9% of all transcripts in the marine unicellular cyanobacterium *Crocosphaera* (Hewson *et al.*, 2009), and up to 2% of all proteins in bacterial symbionts of the marine worm *Olavius algarvensis* (Kleiner *et al.*, 2013). However, these studies targeted individual bacterial strains with exceptionally high transposase abundances, while our data show that high transposase expression characterize large segments of the entire Baltic Sea bacterial community. These results in combination with the observed correlation between transposase transcripts and gene abundance (Figure 7d) contradicts the view that high densities of transposases only exist as long as they are mostly inactive, and rather supports a dynamic transposase abundance in bacterial populations (Wagner, 2006). The observed high transcriptional activity may also reflect a strong influence by environmental stimuli. For example, *Roseobacter* transposases were highly overexpressed inside compared to outside a North Atlantic phytoplankton bloom (Wemheuer *et al.*, 2015), and transposase transcription depends on nutrient availability in *Microcystis* (Steffen *et al.*, 2014) and the archaeon *Methanosarcina* (Jäger *et al.*, 2009). Our findings that transposase expression levels differ significantly between IS families furthermore suggests differential transcriptional regulation, and potentially a specificity in terms of which transposases respond to a certain stimuli.

In contrast to the community-level correlation between transposase transcript and gene abundance, the high transposase expression seen in Cyanobacteria was not accompanied by an equally high genomic transposase abundance (Figure 3). While the reason for this is not clear, the most highly expressed transposase family in our data set was IS200/IS605 (Figure 6a), a family that uses an unusual site-specific (3’ to the tetranucleotide TTAC) transposition mechanism (Ton-Hoang *et al.*, 2010). The majority of these transposases were annotated to the filamentous nitrogen-fixing cyanobacterial order Nostocales, in line with such transposases being highly expressed in the Baltic Sea bloom-forming *Nodularia spumigena* in response to high light and oxygen (Kopf *et al.*, 2015). Moreover, an anti-sense transcript originating from an IS200 transposase incapable of transposition, was found to be involved in regulating up to 30 host genes in *Salmonella* (Ellis and Haniford, 2016). Other examples of ‘domestication’ of IS200/IS605 transposase exist where the bacterial hosts uses their properties for cellular functions (Siguier *et al.*, 2014). Possibly, similar phenomena contribute to the high transposase expression in Cyanobacteria, a bacterial group where regulatory RNAs are common (Kopf and Hess, 2015). Interestingly, the IS200/IS605 family also dominates transposase transcription in the transposase-rich Cyanobacteria *Crocosphaera* (Hewson *et al.*, 2009) and *Microcystis* (Steffen *et al.*, 2014). Similar to Nostocales in the Baltic Sea these organisms form blooms and colonies, and *Crocosphaera* also fixes nitrogen. It is therefore tempting to speculate that IS200/IS605 family transposases may have a role in some of these life-history traits.

The transposase occurrence in Baltic Sea *Synechococcus*, corroborates the bacterial community-level results at a lower taxonomic level. Genome size did not explain the high abundance, again suggesting an influence of positive selection factors (that is, environmental variability or stress). That open ocean *Synechococcus* reference strains mostly lack transposases, while coastal strains are more transposase-rich (Table 1) indicate that such factors select for high transposase levels also in other coastal/estuarine environments. However, the highest levels in the Baltic Sea (133 transposases per genome) is considerably higher than any available *Synechococcus* genome (that is, from the *Synechococcus/Prochlorococcus* cluster 5 of the cyanobacterial lineage). Given that in size fractionation via serial filtration smaller cells often get caught on larger filters (Padilla *et al.*, 2015), the *Synechococcus* transposase occurrence in the large size fraction is likely an underestimate of the true numbers (Asplund-Samuelsson *et al.*, 2016). A potential presence of even higher transposase frequencies in a subset of Baltic Sea *Synechococcus* is further underlined by our analyses being based on average abundances in populations while transposase genomic occurrence often vary considerably between even closely related strains (Wagner, 2006; Bench *et al.*, 2011). Furthermore, in the large size fraction, the *Synechococcus* transposase transcript levels (3–7%) are similar to those found in natural populations of *Crocosphaera* (2.2–15.9%) (Hewson *et al.*, 2009), in which transposases are suggested to have an important function in creating genomic variability (Zehr *et al.*, 2007).

Baltic Sea picocyanobacteria have cell diameters that in general are <1 μm (Stal *et al.*, 2003), and therefore should readily pass through the 3.0 μm size filter. Since picocyanobacteria are autotrophic and thus unlikely to benefit from associating to particles, the reason why a substantial fraction (18%) of these are found in the largest size fraction is likely colony formation or cell aggregation. These phenomena have been well documented for picocyanobacteria in limnic waters (Callieri *et al.*, 2012), but also to some extent among marine strains (Masquelier and Vaulot, 2008). In the Baltic Sea, colonial forms are common, but the abundance is variable, ranging from 5 to 70% of the picocyanobacterial populations (Hajdu *et al.*, 2007). Similar to particles, colonies are characterized by close proximity between cells, cyanobacterial and those of associated heterotrophic bacteria (Dziallas and Grossart, 2011), and may therefore allow a higher rate of gene exchange, promoting the higher transposase abundances of *Synechococcus* seen in the large size fraction. Again, an interesting parallel can be made to transposase-rich *Crocosphaera* and *Microcystis*, both being unicellular colony-forming cyanobacteria (Kaneko *et al.*, 2007; Frangeul *et al.*, 2008; Foster *et al.*, 2013). That the colonial/aggregated lifestyle promotes high transposase frequencies is also supported by the lack of significant difference in genome size between *Synechococcus* from the two larger size fractions (Supplementary Information Figure 7).

As high transposase densities often result in genome rearrangements and inactivated genes (Bhaya *et al.*, 2007), such phenomena are likely present also in genomes of transposase-rich Baltic Sea picocyanobacteria. A transposase-enabled higher genomic plasticity would furthermore connect to the high phylogenetic diversity observed among Baltic Sea cyanobacteria (Celepli *et al.*, 2016) and also be expected to result in novel adaptations. Indeed, a pigment gene cluster not seen previously in any sequenced *Synechococcus* genome was recently characterized from a dominant Baltic Sea picocyanobacterial clade (Larsson *et al.*, 2014). This cluster contains genes necessary for generating both green and red pigmentation and in some cases also two transposases, suggesting a role of transposases in the HGT event that formed the cluster (Larsson *et al.*, 2014). In *Staphylococcus* and *Pseudoalteromonas*, the switch between planktonic and biofilm forming phenotypes (phase variation) is regulated via reversible insertion/excision of IS256 family transposases into biofilm-essential genes (Ziebuhr *et al.*, 1999; Valle *et al.*, 2007; Hennig and Ziebuhr, 2008; Perez *et al.*, 2015). Such transposition events are in turn regulated by the global stress response regulator σ^B^ (Valle *et al.*, 2007). Notably, the Baltic Sea IS256 transposases were almost exclusively annotated to *Synechococcus* and were highly expressed in the large size fraction (Figure 6a). Further studies are now needed to determine whether similar stress-induced and transposase-enabled phenotypic variability exists in transposase-rich Baltic Sea picocyanobacteria.

The high transposase levels observed in bacteria in the small size fraction of deep suboxic Baltic Sea waters (Figure 2) corroborate findings from deep ocean waters (DeLong *et al.*, 2006; Konstantinidis *et al.*, 2009). The large average genome size of suboxic water bacteria (Figure 7a; Konstantinidis *et al.*, 2009) may partly explain the observed enrichment. Also our observation of transposase enrichment in the two larger size fractions corroborate earlier findings, that is, of high transposase abundance in particle-associated bacteria in the open ocean (Ganesh *et al.*, 2014). The size fraction distribution of Rhodobacteriales and Rhizobiales (Supplementary Information Figure 3) thus suggests their high transposase content being influenced by particle association. Mechanisms suggested to explain this phenomenon include the environment on particles being more variable and offering increased opportunities for HGT (Stewart, 2013). However, the observed correlation between transposase density and genome size (Figure 7a) suggests that the larger genomes of particle-associated bacteria (Dupont *et al.*, 2014) may also influence their transposase load. While the exact contribution of different factors in promoting high transposase abundance in particle-associated bacteria remains to be determined, it is nonetheless important to consider the effect of these elevated abundances, that is, that such bacteria likely have higher genomic plasticity than free-living bacteria. This is of extra relevance for estuarine waters where particle-associated bacteria constitute a considerably larger proportion of the total bacterial community than in the oligotrophic ocean (Simon *et al.*, 2002). Particle-associated bacteria may furthermore disassociate from particles, thereby influencing gene frequencies in the free-living fraction (Ganesh *et al.*, 2014). In particle-rich waters this effect may be more pronounced, and potentially contributes to the overall elevated transposase levels seen in the Baltic Sea.

## Conclusions

In this first in-depth community-wide analysis of transposase abundance and expression in natural aquatic bacterial populations, we show that bacteria in the brackish waters of the Baltic Sea contain transposase gene abundances similar to levels observed in extreme environments. Possibly, the pronounced environmental variability and/or specific stress factors select for bacteria with a high genomic plasticity. Furthermore, the high transposase gene expression detected contrasts in the prevailing view of a tight regulation of these genes, suggesting a dynamic presence of transposases in bacterial populations. Transposase gene expression was particularly pronounced in Cyanobacteria, indicating additional regulatory roles of transposases in these bacteria, and picocyanobacteria showed an exceptionally high transposase abundance likely influenced by colony formation. Together, our findings support an important role of transposases as modulators of bacterial genomes, promoting adaptive evolutionary processes in dynamic environments such as the Baltic Sea, and also constitute a platform for continued experimental verifications.

## Data availability

The metagenomic data analyzed during the current study are available in the iMicrobe depository under accession number CAM_P_0001109 (http://imicrobe.us/project/view/114), and the metatranscriptomic data in the NCBI GenBank SRA depository under accession number PRJNA320636 (https://www.ncbi.nlm.nih.gov/bioproject/PRJNA320636).

## Figures and Tables

**Table 1:** Number of transposases per genome in, based on the taxonomic annotation of the metagenomic reads, strains most closely related to the Baltic Sea and Skagerrak *Synechococcus* populations

*Strain*	*Transposase count*	*Genome size (Mbp)*	*Transposases/Mbp*	*Strain origin*
*Coastal (Baltic Sea)*				
* Synechococcus* sp. CB0101	15	2,7	5,6	Chesapeake Bay
* Synechococcus* sp. CB0205	9	2,4	3,7	Chesapeake Bay
* Synechococcus* sp. RS9917	22	2,6	8,5	Red Sea
* Synechococcus* sp. WH5701	50	3,0	16,4	Long Island Sound
				
*Marine (Skagerrak)*				
* Synechococcus* sp. BL107	0	2,3	0	Blanes Bay, Mediterranean Sea
* Synechococcus* sp. CC9311	0	2,6	0	California Current
* Synechococcus* sp. CC9902	0	2,2	0	California Current
