# Supplementary material for: High abundance and expression of transposases in bacteria from the Baltic Sea
Source: ISME J. 2017 Jul 21;11(11):2611–23. doi: 10.1038/ismej.2017.114 (PMC5649170; doi:10.1038/ismej.2017.114)

Supplementary Figures

SI Figure 1. Transposase gene abundances in the Pacific Ocean. (A) Transposase genes relative to all bacterial genes (metagenomic reads), and (B) trransposase transcripts relative to all bacterial transcripts (metatranscriptomic reads). Two depths (upper box) at five stations located at increasing distances from the Californian coast (170-730 km) were sampled and the metagenomic samples were fractionated into three size classes (left). Based on metagenomic and metatranscriptomic primary data sets from Dupont et al., 2015.

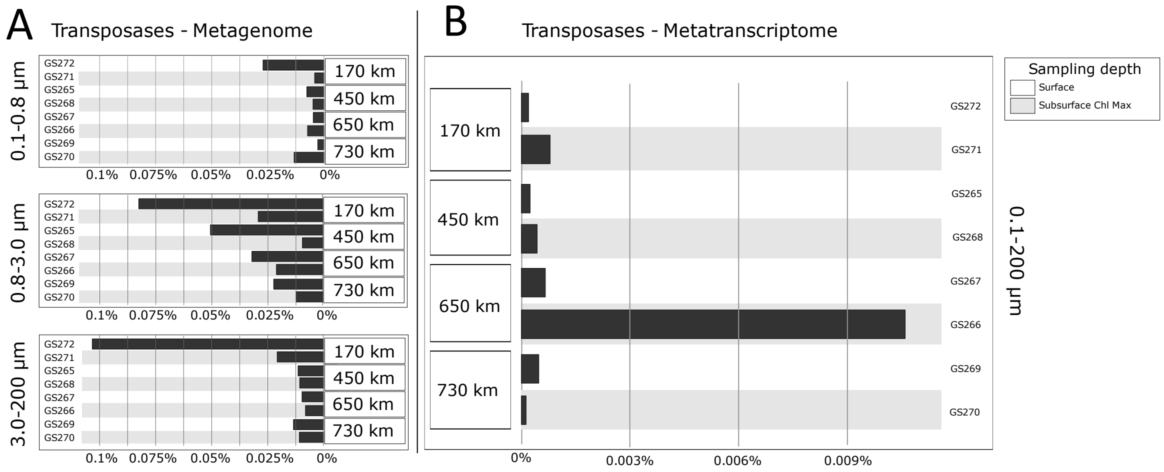

SI Figure 2. Bacterial community composition in the Baltic Sea and adjacent waters. Relative contribution of major bacterial phyla/classes in each fraction of each sample collected in the Baltic Sea, lake Torne Träsk and the marine Skagerrak, based on annotation of metagenomic reads. Colors denote phylum/class (boxes, right).

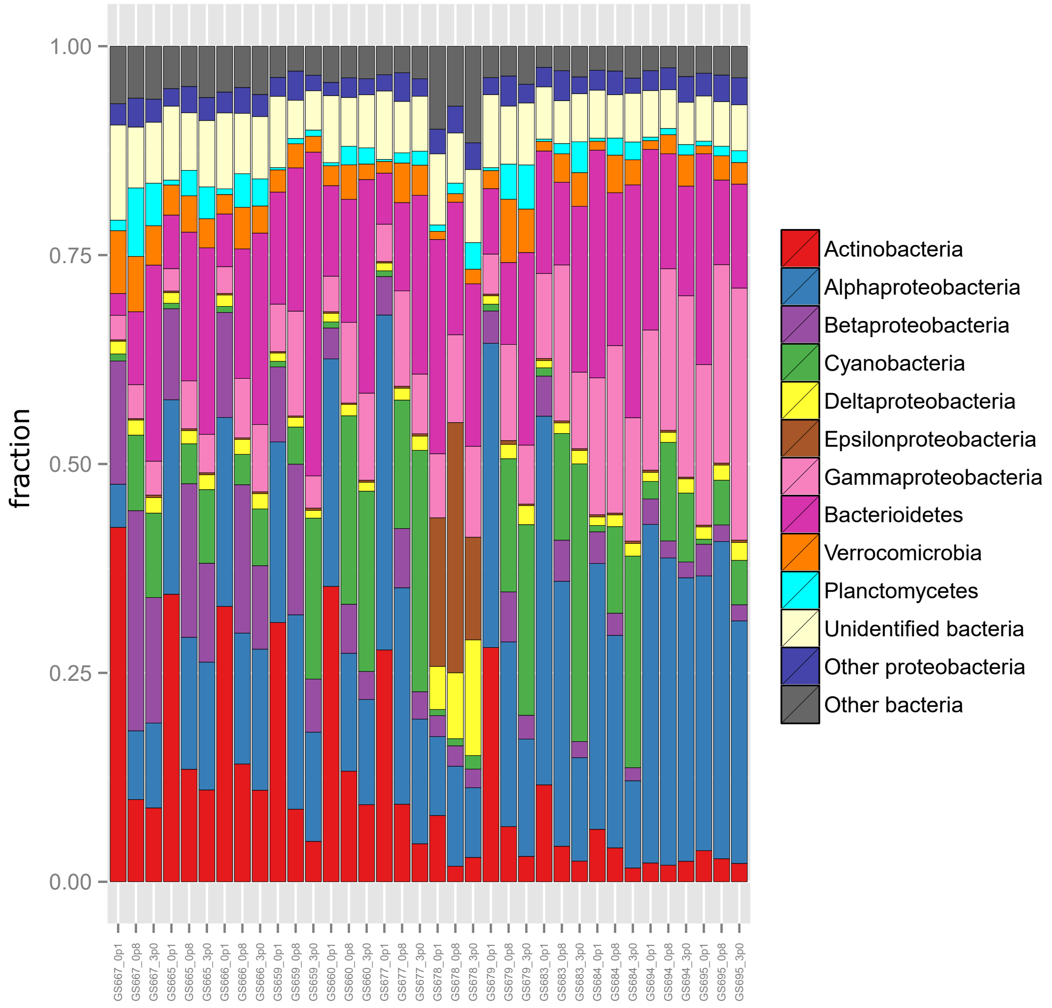

SI Figure 3. **Alphaproteobacterial transposases.** Alphaproteobacterial transposases retrieved from metagenomes along the Baltic Sea transect. The samples are were size fractionated and the colors of the bars represent four major alphaproteobacterial orders (box).

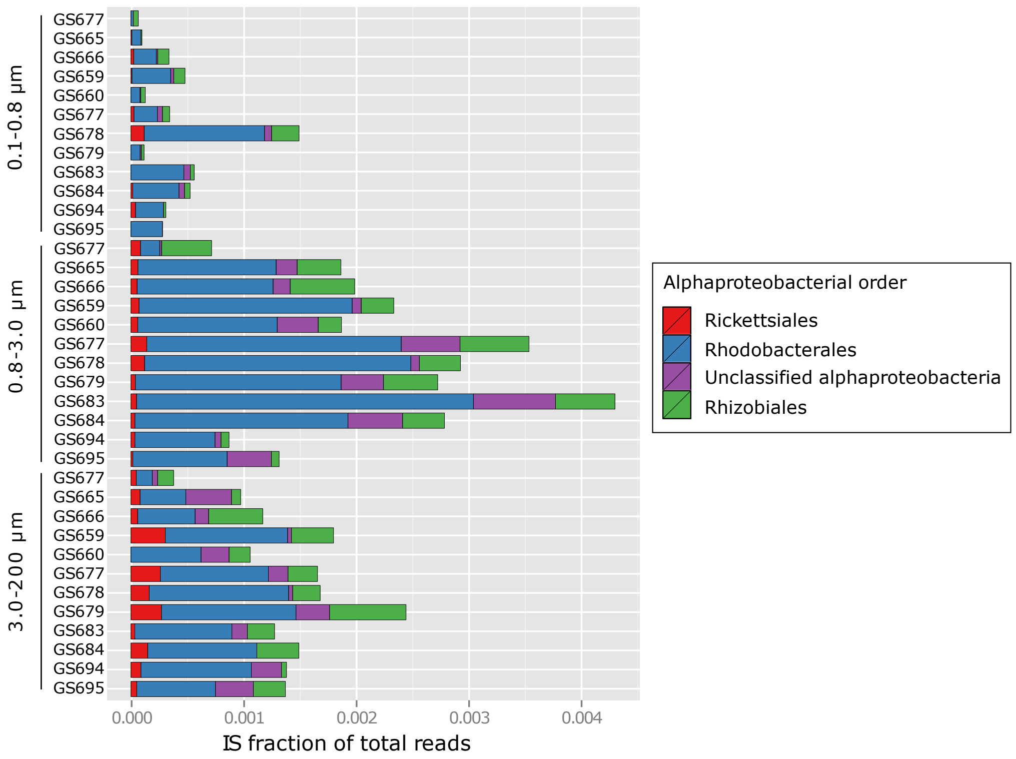

SI Figure 4. Abundance of cyanobacterial orders in the Baltic Sea. Number of metagenomic reads annotated to different cyanobacterial orders relative to all bacterial metagenomic reads in each size fraction of each sample. Cyanobacterial orders are indicated by colors (boxes, right).

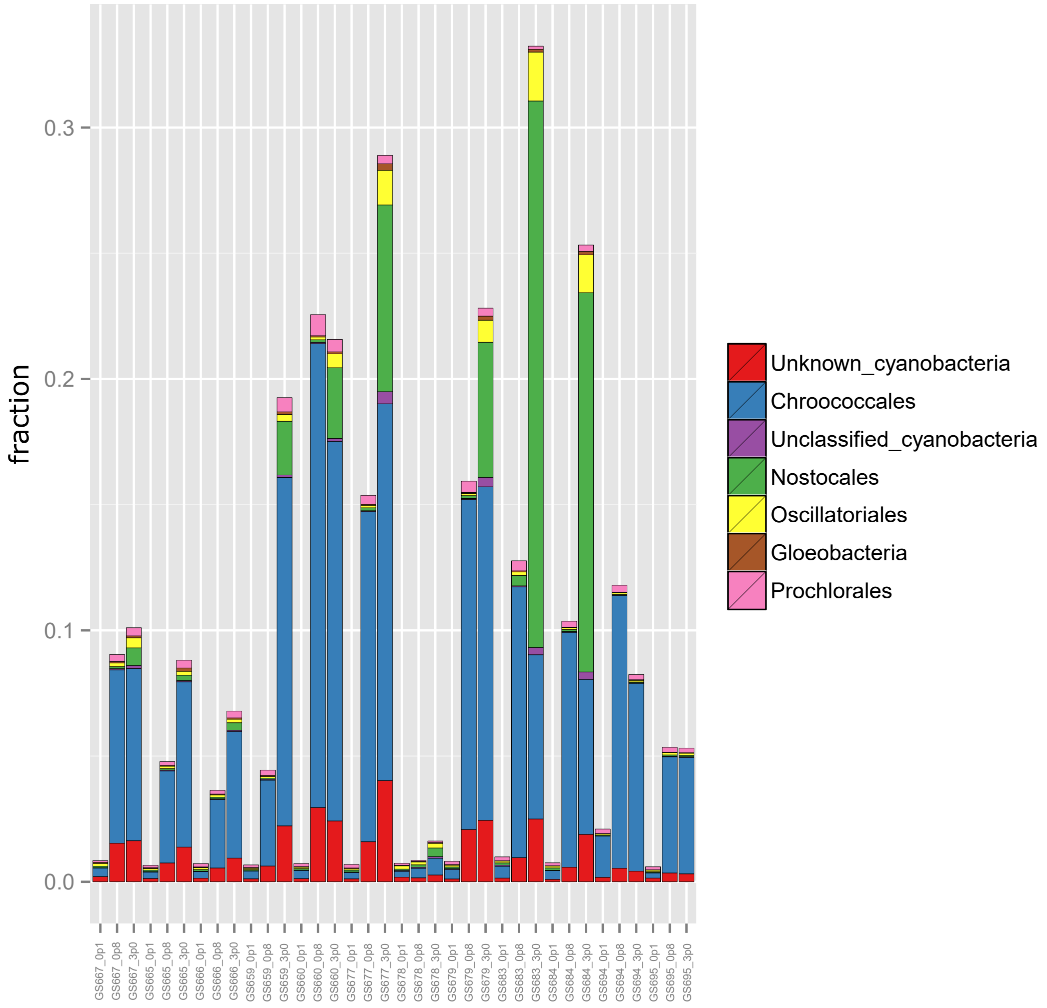

SI Figure 5. Abundance of Chroococcales genera in the Baltic Sea. Number of metagenomic reads annotated to different Chroococcales genera relative to all bacterial metagenomic reads in each size fraction of each sample. Chroococcales genera are indicated by colors (boxes, right).

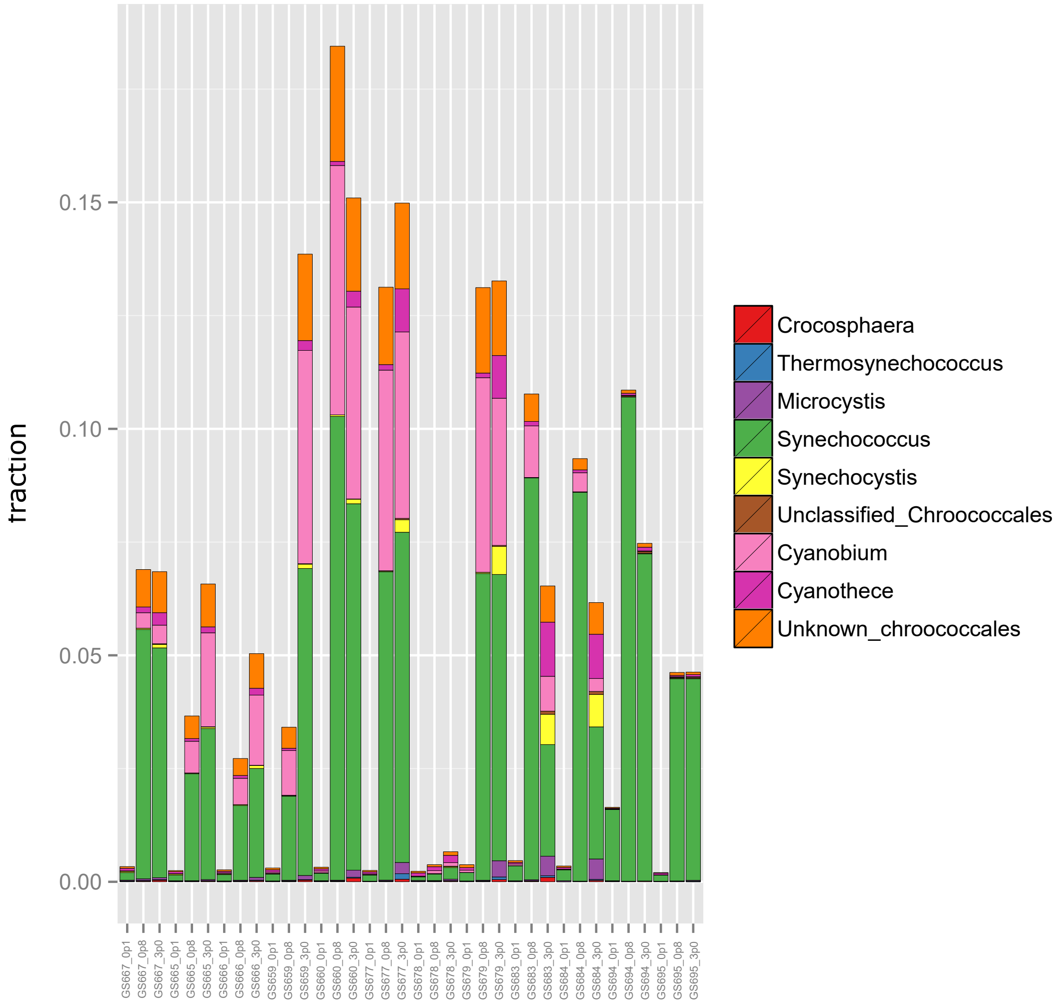

SI Figure 6. *Synechococcus* transposase abundance and expression in the Baltic Sea and Skagerrak. The bars denote the ratio at each station between (A) the number of *Synechococcus* transposase metagenomic reads and all *Synechococcus* metagenomic reads, and (B) the number of *Synechococcus* transposase transcripts and all *Synechococcus* transcripts. Grey bars denote the medium size fractions (0.8-3.0 µm) and black bars the large size fractions (3.0-200 µm). The small size fractions are not shown due to their low numbers of transposase reads/transcripts. No *Synechococcus* transposases were detected in the Skagerrak samples (GS694-95).

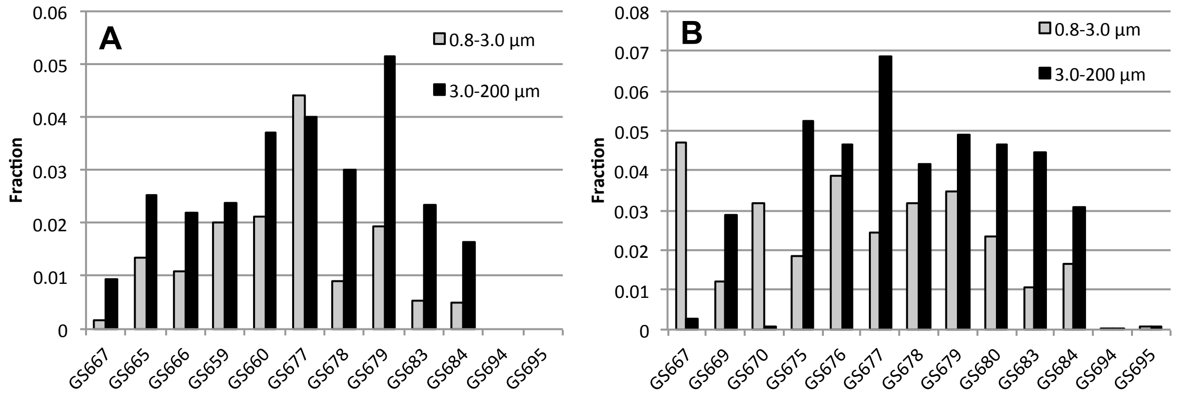

SI Figure 7. *Synechococcus* genome size in different size fractions. As a proxy for genome size, ratio of all metagenomic *Synechococcus* reads to the average abundance of *Synechococcus* 'Single Copy Genes' (SCG) in each sample is shown. No significant difference was found between the medium (0.8-3.0) and large (3.0-200 µm) size fractions.

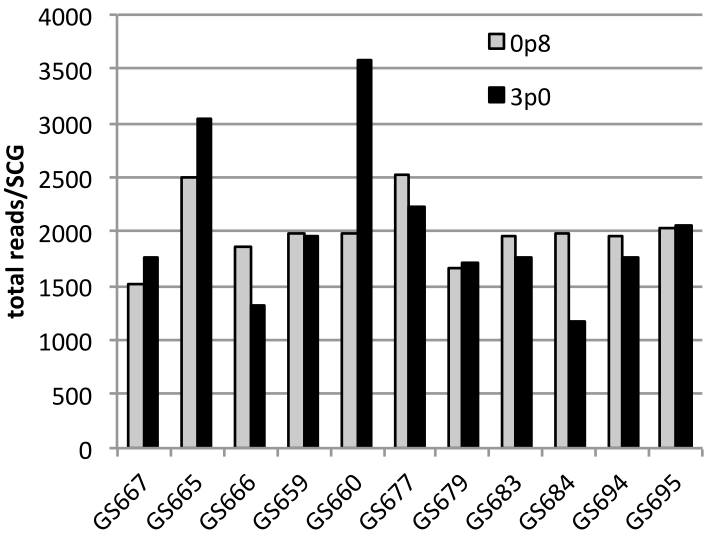

SI Figure 8. *Synechococcus* strain distribution along the sampling transect. The metagenomic contributions by strains, identified via the annotation of the metagenomic samples. *Synechococcus* strans most closely related to WH 5701, CB0205 and CB0101 were common in the Baltic Sea, while *Synechococcus* strains related to strains CC9311, CC9902 and BL107 were more abundant in Skagerrak (GS694-95) and the Danish Straits (GS683-84).

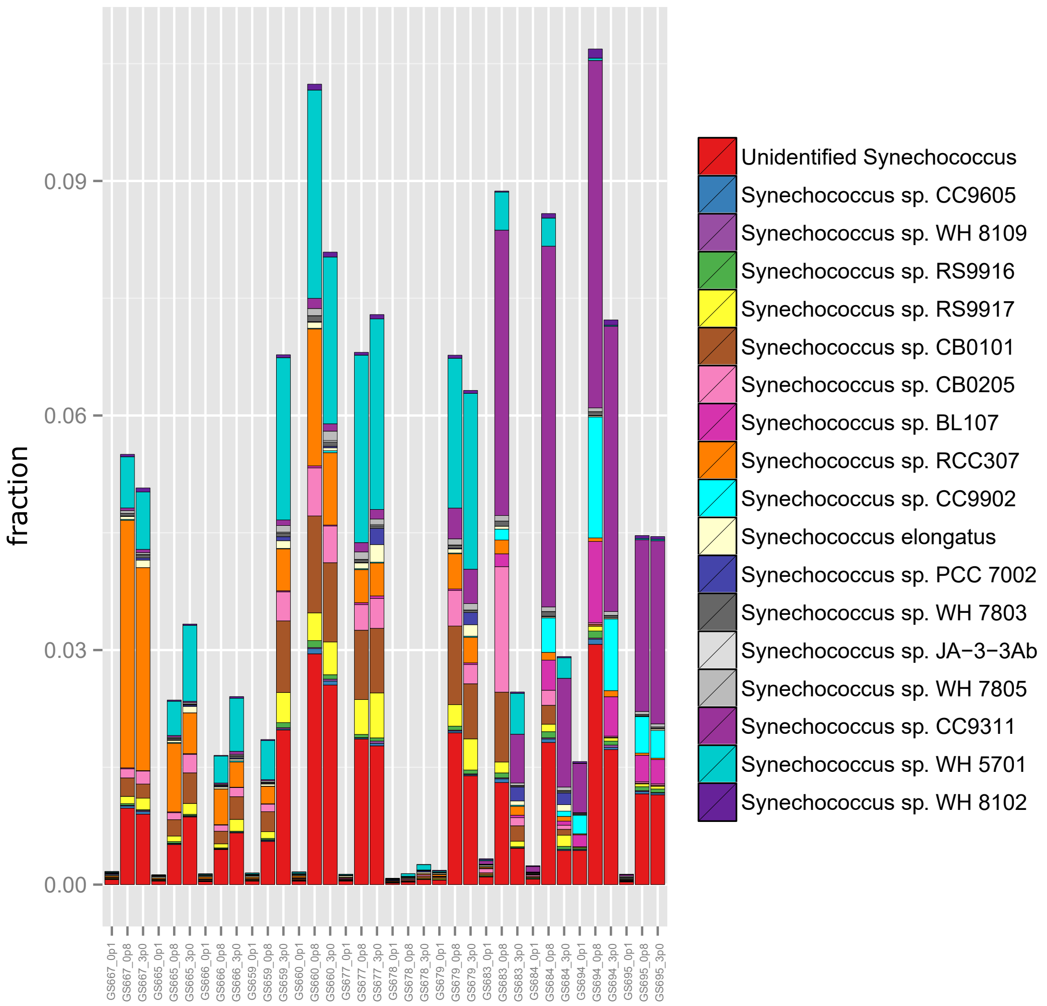

SI Figure 9. Transposase families within the Baltic Sea bacterial transposase population. Bars represent (A) transposase genes as a fraction of total metagenome and (B) transposase transcripts as a fraction of total metatranscriptome for each individual sample. Colors denote the most prevalent IS families.

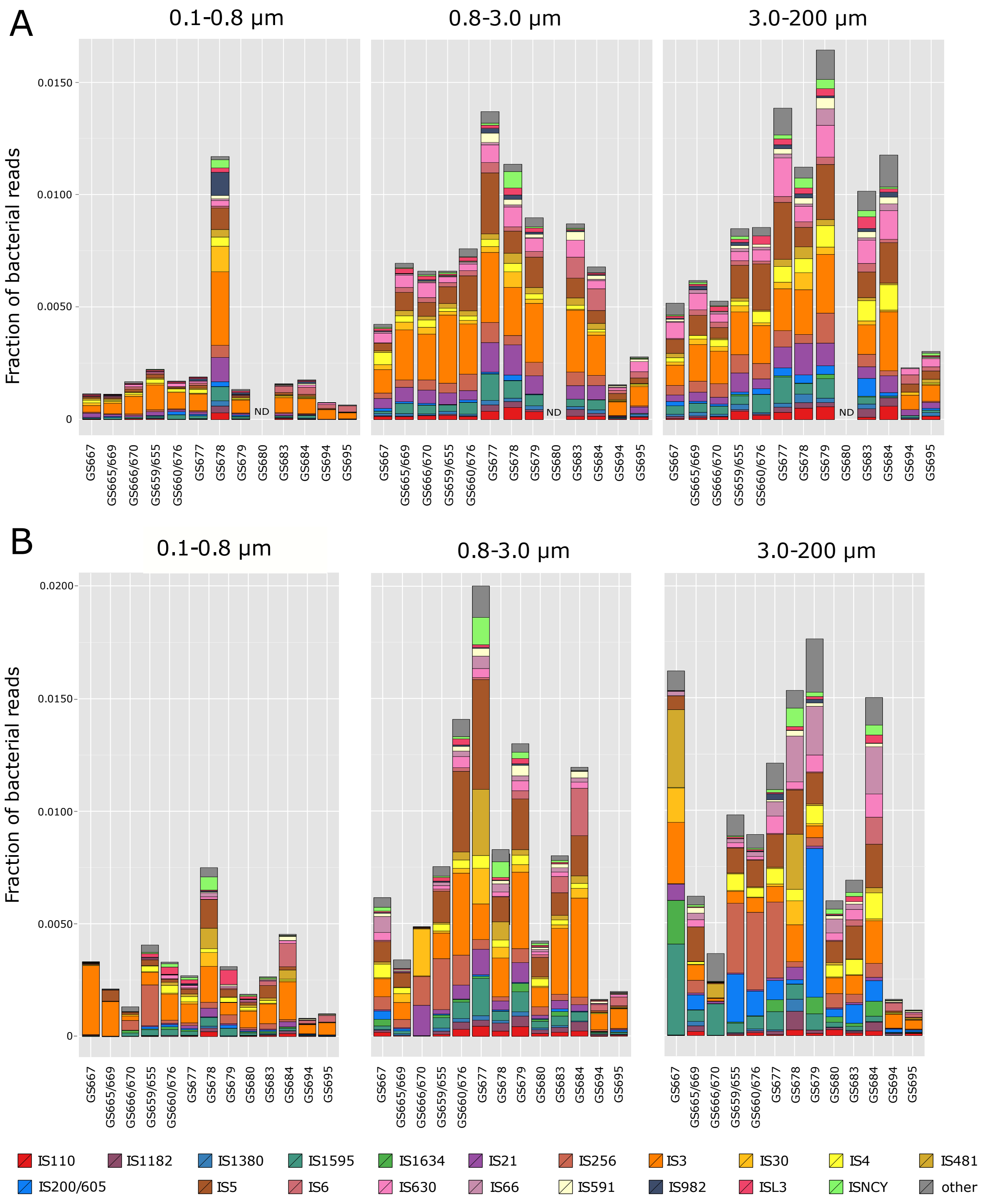

SI Figure 10. Contribution of bacterial phyla to transposase families in the Baltic Sea. Phyla are indicated by color as specified below the graphs. Data for the ten most common transposase families are shown. A-C: Relative abundances in the three size fractions of the meatranscriptomic dataset. D-F: relative abundances in the three size fractions of the metatranscriptomic dataset.

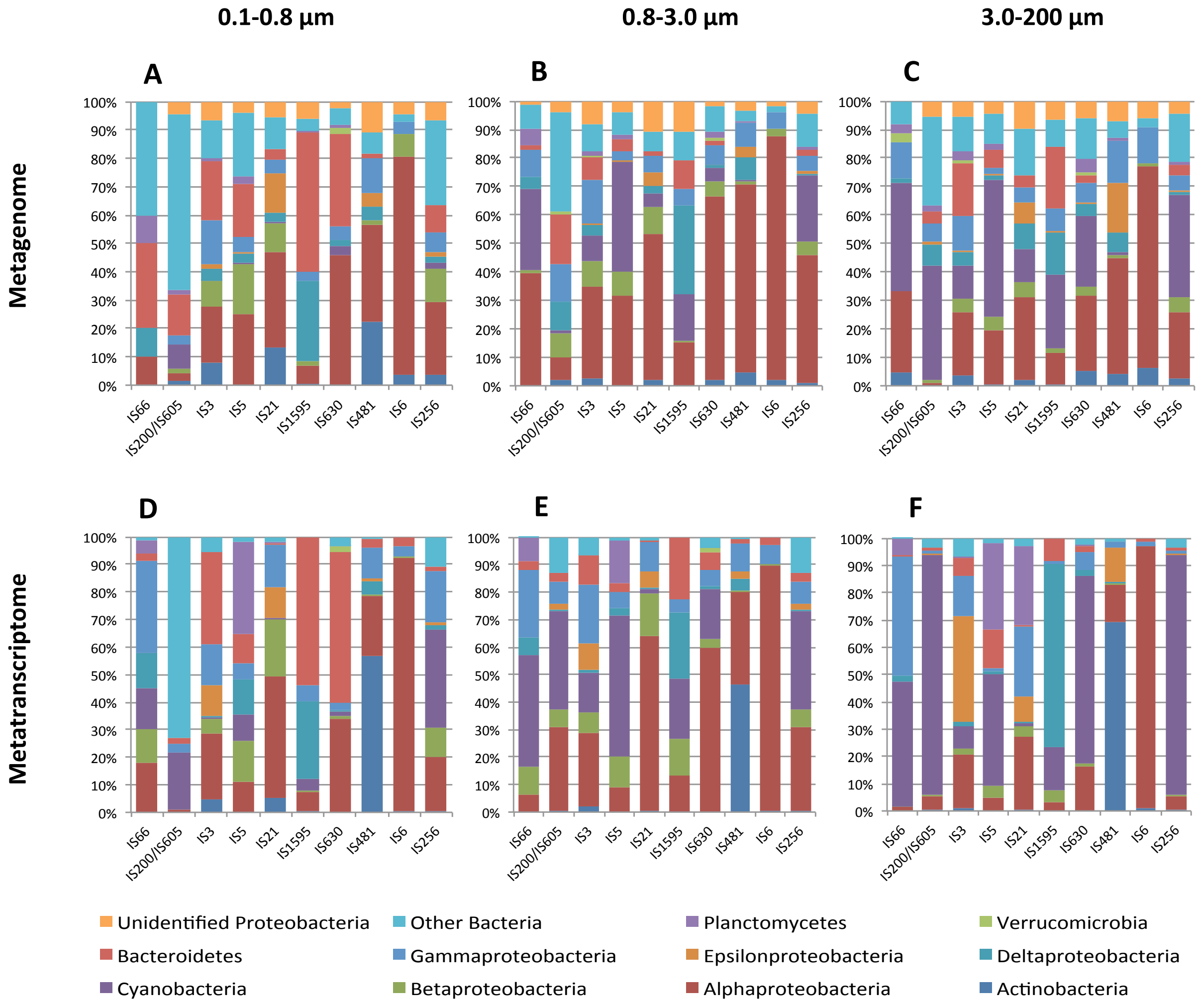

Supplement: Supplementary Figures [file ismej2017114x1.pdf]
